# Calcium-activated chloride channels clamp odor-evoked spike activity in olfactory receptor neurons

**DOI:** 10.1038/s41598-018-28855-3

**Published:** 2018-07-13

**Authors:** Joseph D. Zak, Julien Grimaud, Rong-Chang Li, Chih-Chun Lin, Venkatesh N. Murthy

**Affiliations:** 1 Department of Molecular and Cellular Biology & Center for Brain Science, Harvard University, 16 Divinity Ave, Cambridge, MA 02138 USA; 2 Molecules, Cells & Organisms Program, Harvard University, Cambridge, 02138 MA USA; 3 Johns Hopkins University School of Medicine, Preclinical Teaching Building Room 905A, 725 North Wolfe Street, MD 21205 Baltimore, USA

## Abstract

The calcium-activated chloride channel anoctamin-2 (Ano2) is thought to amplify transduction currents in olfactory receptor neurons (ORNs), a hypothesis supported by previous studies in dissociated neurons from *Ano2*^*−/−*^ mice. Paradoxically, despite a reduction in transduction currents in *Ano2*^*−/−*^ ORNs, their spike output for odor stimuli may be higher. We examined the role of Ano2 in ORNs in their native environment in freely breathing mice by imaging activity in ORN axons as they arrive in the olfactory bulb glomeruli. Odor-evoked responses in ORN axons of *Ano2*^*−/−*^ animals were consistently larger for a variety of odorants and concentrations. In an open arena, *Ano2*^*−/−*^ animals took longer to approach a localized odor source than *Ano2*^*+/+*^ animals, revealing clear olfactory behavioral deficits. Our studies provide the first *in vivo* evidence toward an alternative or additional role for Ano2 in the olfactory transduction cascade, where it may serve as a feedback mechanism to clamp ORN spike output.

## Introduction

Each subtype of olfactory receptor neuron (ORN) converges on a few locations in the olfactory bulb (OB), and therefore serves as a distinct input channel to the brain. ORNs generate electrical signals, in the form of action potentials (spikes), that are interpreted by postsynaptic cells in the OB^1^, including local and projection cells. The series of molecular events that coordinate olfactory transduction and spike generation have been well-delineated, yet much remains unknown about how each individual step in the transduction cascade contributes to overall ORN excitation and output. Specifically, the role of the calcium-activated chloride channel anoctamin-2 (Ano2; also called TMEM16B) remains controversial: many studies point toward its role in massively amplifying ORN transduction currents^2–8^, while paradoxically limiting ORN spike output^9^. In addition, there is conflicting evidence for its importance in olfactory behaviors^8–10^.

Odorants drawn into the nasal cavity bind to odorant receptors (ORs) on the cilia of ORNs. A large number (but not all) of these ORs are G-protein coupled receptors^11–13^, which trigger an intracellular signaling cascade leading to the opening of cyclic nucleotide-gated channels and a net inward flux of Na^+^ and Ca^2+^ ions. The increased intracellular abundance of Ca^2+^ then activates the calcium-activated chloride channel (CaCC) Ano2^9,14,15^. As a result of the elevated intracellular Cl^−^ concentration in ORNs and lower Cl^−^ concentration extracellularly in the nasal epithelium^16–18^, negatively-charged Cl^−^ anions flow outward^16,18^, resulting in an amplification of ORN membrane depolarizations^19^. As much as 90% of the total ORN transduction current may be mediated by Ano2^3,7^, making it a critical component in the sensory transduction pathway that leads to OB input.

Surprisingly, despite its large contribution to the generation of olfactory transduction currents, recent work has suggested that Ano2 is not necessary for odor detection and discrimination^8^. Even the nature of the contribution of Ano2 to ORN activity has become uncertain, since a recent study has suggested that Ano2 may function to limit overall ORN excitability by contributing to a potent depolarization-block of Na^+^ channels^9^. Further investigation of the role that Ano2 plays in olfactory transduction is necessary to understand the precise mechanisms by which odor-evoked excitatory signals are transmitted to the brain.

An additional consideration in the function of Ano2 in olfactory transduction is its expression pattern within ORNs. There is clear evidence that, in addition to being expressed at their cilia^11,20^ within the olfactory epithelium, Ano2 is also abundantly expressed in ORN axons as they terminate in their respective glomeruli^8^. This raises the important possibility that differences between the extracellular Cl^−^ concentration in nasal mucosa/epithelium and the brain may result in Ano2 playing distinct roles in olfactory transduction in different sub-cellular compartments.

How Ano2 contributes to ORN spike output in the native environment of an intact animal, especially at the axon terminals in the OB, has not been addressed. Here, we examine the role of Ano2 in the transmission of odor information to the brain by examining stimulus-evoked responses in ORN terminals in the olfactory bulb of mice with and without Ano2.

## Results

### Glomerular odor maps are unaltered in *Ano2*^*−/−*^ animals

The axons of ORNs of a common subtype coalesce at a few glomeruli on the surface of the OB. The fasciculation of ORN axon bundles as they enter their respective glomeruli is an activity dependent process^21–23^ and could in principle be affected by changes in spontaneous, as well as overall ORN excitability in mice lacking Ano2. Past studies provide conflicting evidence as to whether Ano2 is required for proper targeting of ORNs to glomeruli, with one study reporting that glomerular positioning and number is unaffected in *Ano2*^*−/−*^ mice for two ORN receptor subtypes^8^, while yet another study found an increase in the number of glomeruli incorporating axons of ORNs that express the I7 receptor^9^. We tested whether loss of Ano2 alters functional glomerular maps in the OB.

We crossed heterozygous *Ano2*^*−/+*^ mice^24^ with a mouse strain that expresses the Ca^2+^ indicator GCaMP3 in all ORNs (OMP-GCaMP)^25^ to obtain two groups of mice, *Ano2*^*−/−*^*/OMP-GCaMP3* and *Ano2*^+/+^*/OMP-GCaMP3*. We refer to these two groups as *Ano2*^*−/−*^ and *Ano2*^+/+^ for simplicity throughout this study. First we used wide-field epifluorescence imaging to obtain functional maps of activated glomeruli for seven monomolecular odors by measuring odor-evoked increases in GCaMP fluorescence at ORN axon terminals^26^. These odors (see Methods) were selected to activate a diverse number and range of glomeruli on the dorsal surface of the mouse OB^27^.

**Figure 1:**
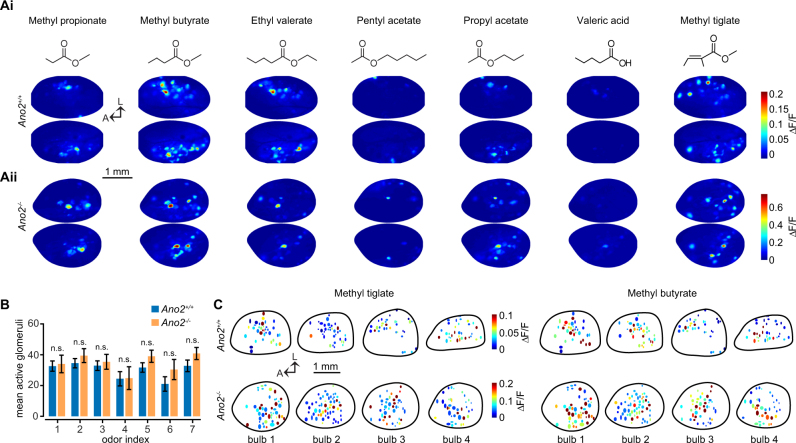
Functional maps of ORN activity. **Ai-Aii**. Example functional maps for seven unique odors in a representative *Ano2*^+/+^ (top) and *Ano2*^−/−^ (bottom) animal. Molecular structures of the odors in the panel are depicted above. (**B)** Number of glomeruli per bulb (n = 11 *Ano2*^+/+^ bulbs, n = 5 *Ano2*^*−/−*^ bulbs) responding to each of the seven odors above ROC threshold (threshold = 0.005 ΔF/F; p > 0.05, Wilcoxon rank-sum test with Bonferroni multiple comparison correction). (**C)** Example functional maps from four *Ano2*^+/+^ and *Ano2*^*−/−*^ bulbs each for two different, but related odors. Glomeruli are represented as pseudocolored ellipses. The position of each ellipse reflects the location of a glomerulus within one olfactory bulb hemisphere. The black line designates the boundary of the imaged region of each olfactory bulb.

We compared the total number of glomeruli at each bulb that responded to each odor in our panel for *Ano2*^+/+^ and *Ano2*^*−/−*^ animals. We used receiver operating characteristic (ROC) analysis to determine a threshold to define responsive glomeruli, by comparing the response distribution obtained from all glomerulus-odor pairs to a noise distribution obtained from interleaved blank odor trials in each experiment. An area under the receiver operating curve analysis was performed and the lowest threshold yielding 10 responses for every no odor response was chosen. (threshold = 0.005 ∆F/F; Supplemental Fig. 1). There was no significant difference between the number of active glomeruli per OB in *Ano2*^+/+^ and *Ano2*^*−/−*^ animals for any of the odors (Fig. 1B; p > 0.05, Wilcoxon rank-sum test with Bonferroni multiple comparison correction).

Although our experiments were not designed to identify specific glomeruli, we can nevertheless discount large scale glomerular duplication in *Ano2*^*−/−*^ animals, for example due to a loss of targeting specificity and mixing of ORN axons within individual glomeruli^28^. We also cannot discount small-scale redistribution of glomerular positioning, which is unlikely to exceed expected animal-to-animal variance described previously in control animals^26,28^. Together, these results indicate ORN maps are largely conserved in *Ano2*^*−/−*^ animals and confirm previous anatomical studies^8^.

### ORN input to glomeruli is enhanced in *Ano2*^*−/−*^ animals

**Figure 2:**
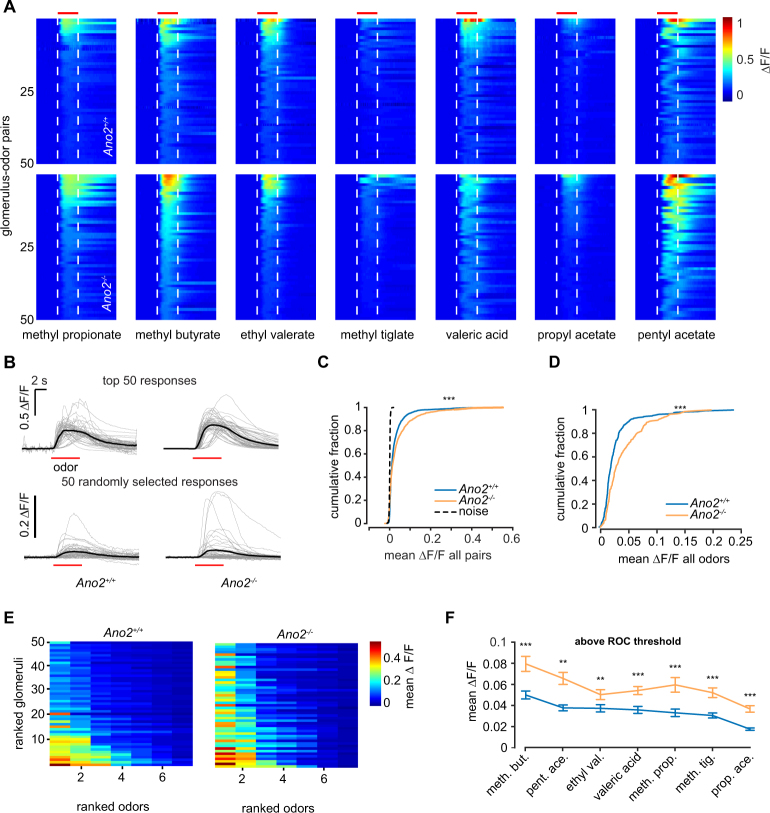
Odor responses in *Ano2*^−/−^ and *Ano2*^+/+^ mice. **(A)** The 50 largest odor-evoked Ca^2+^ signals across all animals for each of the seven odors in both groups. Dashed lines and red bar indicate odor delivery period. Data are sorted by the largest mean response during odor delivery. (**B)** Traces of the 50 largest (top) Ca^2+^ responses for *Ano2*^+/+^ and *Ano2*^*−/−*^ animals across all odors and 50 randomly selected responses (bottom). (**C)** Cumulative distribution of the mean Ca^2+^ response in the odor period across all glomerulus-odor pairs (n = 2093 *Ano2*^+/+^ and 1526 *Ano2*^*−/−*^ glomerulus-odor pairs, p < 0.001 Kolmogorov–Smirnov test). (**D)** Cumulative distribution of the mean Ca^2+^ response across all odors at each glomerulus (n = 299 *Ano2*^+/+^ glomeruli and 218 *Ano2*^*−/−*^ glomeruli, p < 0.001, Kolmogorov–Smirnov test). (**E)** The top 50 glomeruli ranked by mean response across all odors and further ranked by individual odor responses for *Ano2*^+/+^ (right) and *Ano2*^*−/−*^ (left) animals. (**F)** Mean response of all glomeruli responding above ROC-defined threshold (threshold = 0.005 ΔF/F, Wilcoxon rank-sum test with Bonferroni correction, *p < 0.05, **p < 0.01, ***p < 0.001).

We next asked whether odor-evoked responses in individual glomeruli were altered in magnitude in *Ano2*^*−/−*^ animals. From six *Ano2*^+/+^ animals (n = 9 bulbs) and three *Ano2*^*−/−*^ animals (n = 5 bulbs), we identified 34.44 ± 3.48 and 43.60 ± 3.93 glomeruli per bulb, respectively (299 total glomeruli in *Ano2*^+/+^ animals and 218 total glomeruli in *Ano2*^*−/−*^ animals). The 50 largest responses for each odor are shown in Fig. 2A. In addition, traces for the largest responses as well as for randomly chosen responses are shown in Fig. 2B. Our analysis revealed significantly larger odor-evoked responses in ORNs of *Ano2*^*−/−*^ animals than *Ano2*^+/+^ animals (n = 2093 *Ano2*^+/+^ and 1526 *Ano2*^*−/−*^ glomerulus-odor pairs, p < 0.001 Kolmogorov–Smirnov test; Fig. 2C). This difference was observed both when responses of each glomerulus were averaged across all seven odors (n = 299 *Ano2*^+/+^ glomeruli and 218 *Ano2*^*−/−*^ glomeruli, p < 0.001, Kolmogorov–Smirnov test), and when responses of all glomeruli for each odor were averaged (Fig. 2D–F).

Our data provide evidence that loss of Ano2 results in enhanced ORN input to OB glomeruli. We also observed that the number of glomeruli responding to an odor was similar in *Ano2*^+/+^ and *Ano2*^*−/−*^ animals. These results are consistent with a scaling mechanism, where Ano2 may function as a negative feedback on ORN excitability and limit the number of action potentials generated in response to odor stimulation.

### Loss of Ano2 does not impact respiration

It is possible that the larger responses to odorants in *Ano2*^*−/−*^ animals is due to faster respiration rates and temporal summation of responses^29^. Other ANO isoforms are expressed in smooth muscle tissue and may regulate the excitability of the diaphragm and the airway^30–33^, thereby altering normal breathing rhythms in *Ano2*^*−/−*^ animals. We recorded the respiration rate of *Ano2*^+/+^, *Ano2*^*−/−*^, and C57BL/6 J mice using a thermocouple placed in front of the animal’s nose, under anesthesia conditions consistent with our previous experiments. We first validated the reliability of the external thermocouple for respiration tracking by comparing it to a well-established method, intranasal cannulas (Supplemental Fig. 2). Upon validation, we chose to record respiration using the non-invasive thermocouple to mitigate any effects of damaging the nasal cavity through cannula implantation.

**Figure 3:**
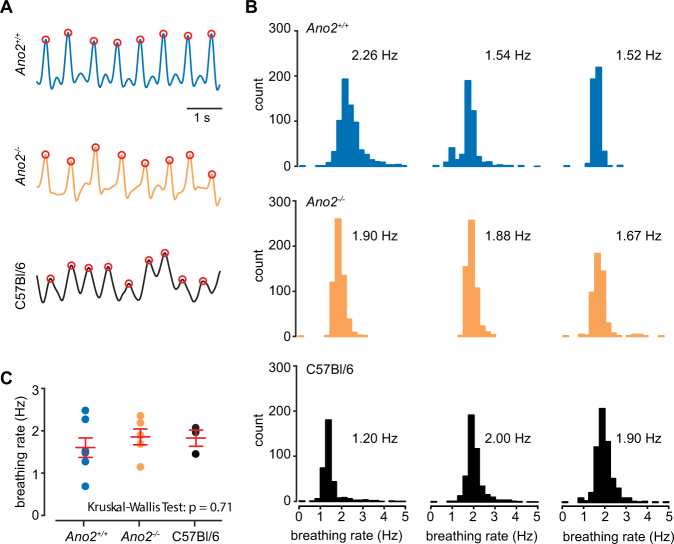
Loss of Ano2 does not impact respiration rate. (**A)** Example respiration traces from a *Ano2*^+/+^, *Ano2*^*−/−*^ and C57BL/6 J animals recorded with a thermocouple placed near the animal’s nose (see Supplemental Fig. 2 for technique validation). (**B)** Histograms of the instantaneous respiration frequency in a 5-minute window from three representative animals from each group. Mean instantaneous frequency is displayed next to each plot. (**C)** Mean instantaneous frequency for all animals in each group. Red bars denote mean and standard error across all animals (p = 0.71, Kruskal-Wallis test).

Robust respiration signals could be recorded in anesthetized mice (n = 6 *Ano2*^+/+^, 7 *Ano2*^*−/−*^, 3 C57BL/6 J; Fig. 3A–B for examples). We included C57BL/6 J animals in our comparison to rule out any genetic background effects^34^. Despite slight animal-to-animal variability, we found that across all groups, there was no statistical difference in the overall respiration frequency (mean frequency: 1.60 ± 0.17 Hz *Ano2*^+/+^, 1.86 ± 0.23 Hz *Ano2*^*−/−*^, 1.77 ± 0.19 Hz C57BL/6 J, p = 0.71 Kruskal-Wallis test; Fig. 3C). The small, statistically insignificant increase in respiration rate we observed in *Ano2*^*−/−*^ animals (16.3% increase) is insufficient to account for the larger Ca^2+^ responses obtained in our previous imaging experiments (p < 0.001, Kolmogorov–Smirnov test).

We conclude that anesthetized *Ano2*^*−/−*^ animals do not breathe with increased frequency and that the larger Ca^2+^ signals we observed are not due to enhanced respiration rate.

### Multiphoton imaging in *Ano2*^−/−^ animals

**Figure 4:**
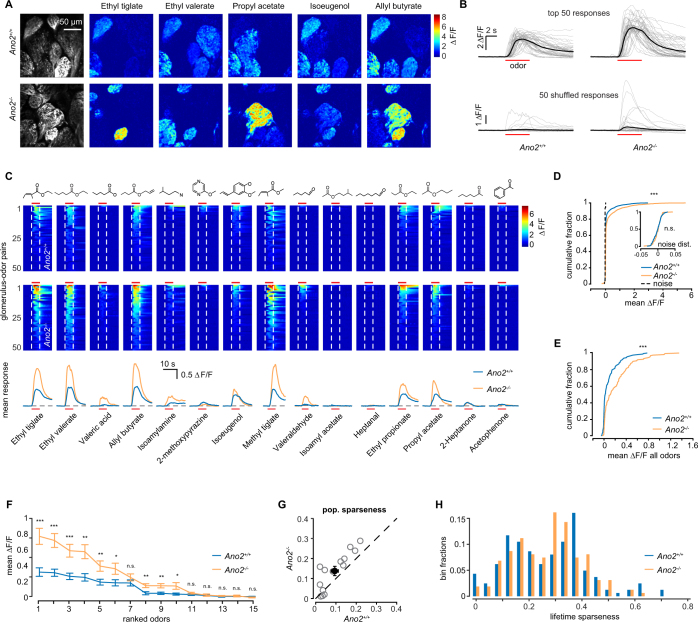
Calcium responses in ORNs measured with multiphoton microscopy. (**A)** Example multiphoton-acquired images of glomeruli from an example *Ano2*^+/+^ and *Ano2*^*−/−*^ animal, as well as example ΔF/F responses for five selected odors. Mean images from 20 frames preceding and during the odor delivery period were used. (**B)** Traces of the 50 largest (top) Ca^2+^ responses for *Ano2*^+/+^ and *Ano2*^*−/−*^ animals across all odors and 50 randomly selected responses (bottom). (**C)** The 50 largest odor-evoked Ca^2+^ signals across all animals for each of 15 odors. Dashed line denotes odor onset and offset and red bar indicates odor duration. Molecular structures are depicted above. Bottom, mean response time course for each odor. (**D)** Cumulative distribution of the mean Ca^2+^ response in the odor period across all glomerulus-odor pairs (n = 2430 *Ano2*^+/+^ and 2415 *Ano2*^*−/−*^ glomerulus-odor pairs, p < 0.001, Kolmogorov–Smirnov test). Inset, distribution of blank odor trial responses used to determine ROC threshold (threshold = 0.02 n = 162 *Ano2*^+/+^ and 161 *Ano2*^*−/−*^ glomeruli, p > 0.05, Kolmogorov–Smirnov test). (**E)** Cumulative distribution of the mean Ca^2+^ response across all odors at each glomerulus (n = 162 *Ano2*^+/+^ and 161 *Ano2*^*−/−*^ glomeruli, p < 0.001, Kolmogorov–Smirnov test). (**F)**. Mean response of all glomeruli responding above threshold for each odor (Wilcoxon rank-sum test with Bonferroni correction, *p < 0.05, **p < 0.01, ***p < 0.001). (**G)** Scatter plot of population sparseness for each odor. Mean across all odors is the filled black circle (mean sparseness = 0.09 ± 0.02 in *Ano2*^+/+^ and 0.13 ± 0.02 in *Ano2*^*−/−*^, p = 0.002, Wilcoxon sign-rank test). (**H)** Histogram of lifetime sparseness across all glomeruli (mean sparseness = 0.28 ± 0.01 in *Ano2*^+/+^ and 0.28 ± 0.01 in *Ano2*^*−/−*^, p = 0.89, Wilcoxon rank-sum test.

Due to the low resting fluorescence of GCaMP3 we were unable to identify glomeruli that did not respond to at least one of the seven odors in our panel using an epifluorescence microscope. We used multiphoton microscopy to overcome this limitation and were able to visualize all glomeruli, independent of their responsiveness (Fig. 4A). We also expanded our odor panel size to 15 odors to activate a wider range of glomeruli. The optical sectioning facilitated by multiphoton microscopy also allowed us to exclude any effects arising from the activity of *en passant* axons that could be detected using our epifluorescence setup. As a result of significant out-of-focus fluorescence in wide-field imaging, signal contamination could arise from Ca^2+^ activity in axons that pass above inactive glomeruli.

Across 162 *Ano2*^+/+^ and 161 *Ano2*^*−/−*^ glomeruli from five animals each, we found that ORNs in *Ano2*^*−/−*^ animals responded with significantly larger Ca^2+^ transients (Fig. 4B–F), whether comparing individual glomerulus-odor pairs (n = 2430 *Ano2*^+/+^ and 2415 *Ano2*^*−/−*^ glomerulus-odor pairs, p < 0.001, Kolmogorov–Smirnov test; Fig. 4D), or mean response across all odors (n = 162 *Ano2*^+/+^ and 161 *Ano2*^*−/−*^ glomeruli, p < 0.001, Kolmogorov–Smirnov test; Fig. 4E). The 50 largest overall responses for each group are displayed in Fig. 4B (mean of all responses above threshold: 38.8 ± 0.02% ∆F/F in *Ano2*^+/+^ and 57.5 ± 0.03% ∆F/F in *Ano2*^*−/−*^). When considering each odor individually, in 9 out of 15 odors we observed significantly larger Ca^2+^ responses in *Ano2*^*−/−*^ animals (Fig. 4H, Wilcoxon rank-sum test with Bonferroni correction).

We next compared the kinetics of the Ca^2+^ responses in *Ano2*^+/+^ and *Ano2*^*−/−*^ animals. Due to the improved signal to noise ratio and optical sectioning of multiphoton microscopy, we were able to compare the responses of individual trials rather than the means of replicates from the same glomerulus. This procedure reduced the influence of respiration, which dictates the response onset. We first characterized the rise time. On average, *Ano2*^*−/−*^ animals responded with a slightly faster rise time than *Ano2*^+/+^ animals (2.35 ± 0.05 s in *Ano2*^+/+^, n = 2614, and 2.25 ± 0.05 s in *Ano2*^*−/−*^, n = 3393, p < 0.001 Kolmogorov–Smirnov test). We also found that the odor responses in *Ano2*^*−/−*^ animals decayed at a slightly faster rate than *Ano2*^+/+^ animals (decay time constant = 4.74 ± 0.08 s in *Ano2*^+/+^, n = 2376, and 4.51 ± 0.06 s in *Ano2*^*−/−*^, n = 3155, p = 0.004 Kolmogorov–Smirnov test). Our data indicate that Ano2 deletion may result in spike generation in ORNs within a narrower window following stimulus delivery, resulting in both a quicker rise and decay in Ca^2+^ responses. However, the slow timescale of GCaMP3 and our population imaging approach leave open the possibility that more dramatic differences may be observed on the single cell level.

Lastly, we compared sparsity of glomerular responses. We calculated the population sparseness (see Methods) to compare the fraction of activated glomeruli across all animals for each odor. Population sparseness is related to the fraction of glomeruli that are activated by a given odor stimulus, with values near one indicating uniform activity across all glomeruli and values near zero indicating highly selective responses across all observed glomeruli. We found that a larger fraction of glomeruli responded in *Ano2*^*−/−*^ animals (population sparseness measure: 0.09 ± 0.02 in *Ano2*^+/+^ and 0.13 ± 0.02 in *Ano2*^*−/−*^, p = 0.002, Wilcoxon sign-rank test, Fig. 4G). We found no differences between *Ano2*^+/+^ and *Ano2*^*−/−*^ animals in lifetime sparseness, which quantifies the extent to which a given glomerulus responds to different odor stimuli (lifetime sparseness measure: 0.28 ± 0.01 in *Ano2*^+/+^ and 0.28 ± 0.01 in *Ano2*^*−/−*^, p = 0.89, Wilcoxon rank-sum test, Fig. 4H). If all odors activate the observed glomerulus uniformly, the lifetime sparseness measure will be close to one, and if a glomerular response is specific to only a small number of odors, the measure will be close to zero. Together these results indicate that ORNs in *Ano2*^*−/−*^ animals are indeed more sensitive to odor stimulation, but the breadth of their odor tuning is unchanged. Furthermore, the fact that we observed no difference in odor tuning further argues against the possibility that glomeruli in *Ano2*^*−/−*^ animals receive heterogeneous innervation from multiple ORN subtypes. We also found no evidence for heterogeneous responses within individual glomerular regions of interest.

### ORNs are more strongly excited by odors across a range of concentrations

What accounts for the larger fraction of activated glomeruli in *Ano2*^*−/−*^ animals? One possibility is that the signal-to-noise ratio afforded by multiphoton microscopy allowed us to identify weak responses arising from odor-receptor binding in *Ano2*^*−/−*^ animals that are sub-threshold for Ca^2+^ signal generation in *Ano2*^+/+^ animals. Conversely, given our previous results, another explanation for the increased Ca^2+^ signal magnitude in *Ano2*^*−/−*^ animals is that in response to high odor concentrations, ORNs are able to maintain firing due to a reduction in depolarization induced Na^+^ inactivation driven by the amplifying current through Ano2. However, at low odor concentrations, ORNs in *Ano2*^*−/−*^ animals may have weaker responses than ORNs in *Ano2*^+/+^ animals, since it has been shown that current amplification through Ano2 is most potent close to detection threshold^24^. We next investigated whether ORNs in *Ano2*^*−/−*^ animals are more responsive to odors at different concentrations.

**Figure 5:**
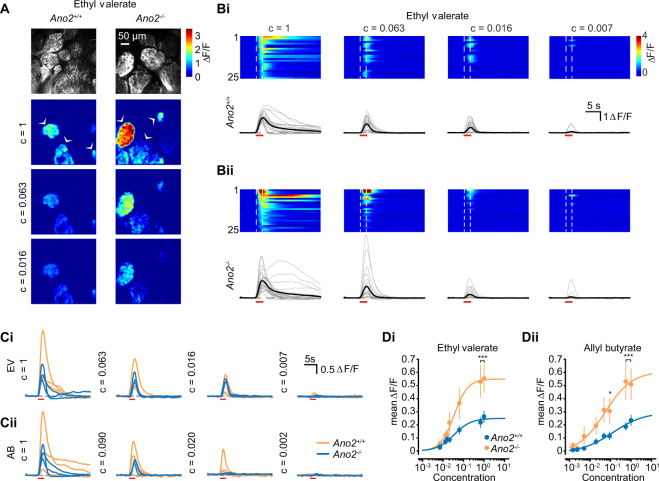
ORN responses in *Ano2*^*−/−*^ animals are enhanced at high odor concentrations. (**A)** Example multiphoton-acquired images of glomeruli and ΔF/F responses at three odor concentrations. Odor concentrations were normalized to the highest concentration (~10% v/v) using a photoionization detector (see Supplemental Fig. 4). (**Bi-ii)** Examples of the 25 largest responses to the highest concentration of Ethyl valerate followed through four other concentrations for *Ano2*^+/+^ (top) and *Ano2*^-/-^ (bottom) animals. Individual traces are displayed below. Dashed line denotes odor onset and offset, red bar indicates odor period. (**Ci-ii**) Example traces from three glomeruli in part A, identified by arrowheads followed through four concentrations of Ethyl valerate (EV; top) and Allyl butyrate (AB; bottom) **Di-ii**. Mean response (filled circles) at eight odor concentrations for both odors and sigmoidal fit to each (Wilcoxon rank-sum, *p < 0.05, **p < 0.01, ***p < 0.001).

We used air dilution to alter odor concentration over four orders of magnitude for two odors, Ethyl valerate and Allyl butyrate, and decreased the odor delivery time to two seconds to prevent saturation of ORN responses at the highest concentrations. The relative concentration of each odor experienced by the animal was verified using a photoionization detector and odor concentrations were normalized to the lowest dilution (see Methods; Supplemental Fig. 3). At the strongest odor concentrations, glomerular ORN responses were again enhanced in *Ano2*^*−/−*^ animals for both odors, as well as a mixture of the two (Fig. 5A–D, Wilcoxon rank-sum test). However, somewhat surprisingly, at low concentrations our analysis revealed no pair-wise differences. For low concentrations, the largest responses typically occurred in *Ano2*^*−/−*^ animals, but the vast majority of response were typically within the range of those observed in *Ano2*^+/+^ animals, thereby limiting our ability to statistically differentiate the two groups (Supplemental Fig. 4). We were unable to compare the response amplitude of ORNs at their individual detection thresholds due to an inability to identify specific subtypes of ORNs (expressing a particular odorant receptor) across animals. We also note that Ca^2+^ signals in ORN axons report spike output rather than transduction current amplitudes. Therefore, our results indicate that while Ano2 may play a role in shunting ORN excitation following strong odor stimulation, it may have less of an effect on ORN activity following weak odor input.

### Ano2 deletion increases latency to odor localization

Does increased ORN excitability alter odor detection capabilities of *Ano2*^*−/−*^ animals? Recent studies provide evidence that *Ano2*^*−/−*^ animals exhibit a greater latency to uncover a hidden food-object^9,10^, while another study was unable to find any difference in odor detection and discrimination using a learned behavior^8^. We decided to study innate odor-driven investigation to assess whether *Ano2*^*−/−*^ animals displayed any sensory deficit independent of learned behavior.

**Figure 6:**
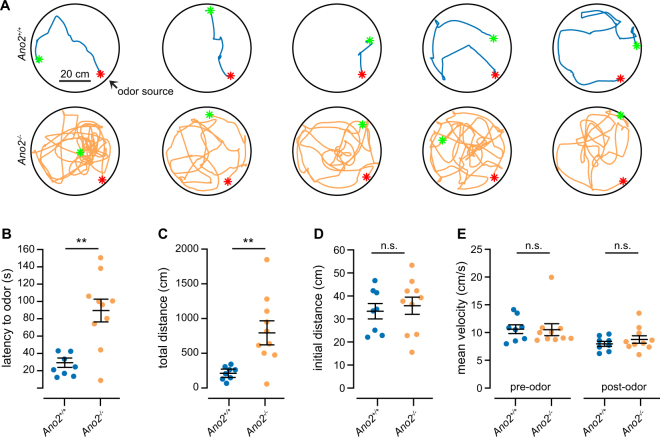
Latency to odor localization is increased in *Ano2*^*−/−*^ animals. (**A)** Examples of five *Ano2*^+/+^ and *Ano2*^*−/−*^ animals tracking to the source of odorized air carrying a 1% dilution of peanut oil. Green and red asterisks mark the initial and final position of each animal. (**B)** Time latency for animals to locate the odor source (n = 8 *Ano2*^+/+^ and 10 *Ano2*^*−/−*^ animals, p = 0.003, Wilcoxon rank-sum test). (**C)** Total distance traveled before finding the odor source across all animals (p = 0.002, Wilcoxon rank-sum test). (**D)** Initial distance from odor source (at odor onset) across all animals (p > 0.05, Wilcoxon rank-sum test). (**E)** Mean velocity of each animal prior to and following odor onset (p > 0.05, Wilcoxon rank-sum test).

We investigated the latency to explore odors as an indicator of how easily animals can detect odors^9,10^. To minimize experimenter-induced biases, our automated experimental apparatus consisted of a 56 cm diameter circular arena with four air inlets equally spaced around its circumference, as well as a vacuum in its center to balance air inflow and outflow. Under infrared illumination, mice were allowed to explore the arena space for 10 minutes, after which odorized air was delivered through one of the inlets. We then measured the latency and path taken by each animal to investigate the odor source, as determined by the animal approaching the odorized air inlet within 1 cm (Fig. 6A).

We used the known appetitive odor peanut oil^35^ diluted to the same concentration as we used in our imaging experiments (1% in mineral oil). Consistent with previous reports^9,10^, across 8 *Ano2*^+/+^ and 10 *Ano2*^*−/−*^ animals, we found that *Ano2*^*−/−*^ animals required significantly more time to locate the odor source (*Ano2*^+/+^  = 29.28 ± 5.75 s, *Ano2*^*−/−*^ = 94.71 ± 16.47 s, p = 0.003, Wilcoxon rank-sum test; Fig. 6B). *Ano2*^*−/−*^ animals also traveled a significantly longer distance before ultimately arriving at the odor source (*Ano2*^+/+^  = 178.41 ± 28.46 cm, *Ano2*^*−/−*^ = 822.43 ± 175.95 cm, p = 0.002, Wilcoxon rank-sum test; Fig. 6C) Because the odor onset occurred independently of the animal location in the arena, we calculated the initial starting distance from the odor source and observed no differences in their mean initial positions (*Ano2*^+/+^  = 30.71 ± 4.68 cm, *Ano2*^*−/−*^ = 37.78 ± 3.58 cm, p = 0.002 Fig. 6D, p > 0.05, Wilcoxon rank-sum test). At the same time, we observed no differences in the locomotor activity of *Ano2*^*−/−*^ animals as measured by their mean velocity both prior to and following odor delivery (Fig. 6E, p > 0.05, Wilcoxon rank-sum test). These behavioral data suggest a puzzling dissociation between the increased responses to odorants in *Ano2*^*−/−*^ animals and the longer latency to locate the source of an appetitive odor.

## Discussion

Our study presents direct evidence in freely-breathing mice that Ano2, despite its role in amplifying transduction currents in ORNs, limits their overall excitation and input to the OB *in vivo*. Our results are in agreement with recent *in vitro* measurements of ORN spike output in *Ano2*^*−/−*^ animals^9^ and further point towards a dual functionality of Ano2 in ORN excitability whereby it both amplifies transduction currents and limits spike output.

### Glomerular maps and respiration

Loss of Ano2 in mice could lead to more general changes that might be confounding factors that undermine conclusions about sensory transduction and coding. First, absence of Ano2 might alter the anatomical organization of glomerular maps in the OB. In particular, spontaneous activity in ORNs is known to play an important role in ORN fasciculation and glomerular emergence^21–23^. Any differences in spontaneous activity between *Ano2*^+/+^ and *Ano2*^*−/−*^ animals might lead to disorganized glomerular organization and odor representation. Our results argue against a broad topographical reorganization of ORN inputs to the OB in *Ano2*^*−/−*^ animals based on two factors. First, we found that the number of dorsal glomeruli responding above threshold to a given odor was unchanged and second, we found no difference in the lifetime sparseness of individual glomeruli from *Ano2*^+/+^ and *Ano2*^*−/−*^ animals. Furthermore, glomeruli in *Ano2*^*−/−*^ animals do not appear to receive heterogeneous ORN innervation since responding glomeruli were invariably homogeneous. Our *in vivo* imaging methods restricted us to imaging the dorsal portion of the OB, and we cannot rule out the possibility that ORN targeting is disrupted in the medial, lateral or ventral OB in *Ano2*^*−/−*^ mice.

A second factor that might affect the data on glomerular imaging is the respiration rate. Faster respiration may lead to larger Ca^2+^ signals because of slow time course of axonal Ca^2+^ as well as indicator kinetics. Direct measurement of respiration, however, dispelled this concern – we found no significant change in respiration rate in *Ano2*^*−/−*^ animals. On a methodological note, we also demonstrated that an externally-placed, non-invasive thermocouple is a reliable method to record and measure breathing responses in anesthetized mice. While this method could be valuable for experiments in anesthetized animals, we note that it rapidly loses fidelity when breathing rate increases, as in awake animals (Supplemental Fig. 2E,F).

### Larger odor-evoked responses in *Ano2*^−/−^ animals

The major finding of our study is that the magnitude of the ORN Ca^2+^ responses following odor stimulation was larger in *Ano2*^*−/−*^ animals, with no observable change in the overall response duration. This was confirmed in two different modes of imaging – widefield microscopy that allowed larger regions to be imaged at lower resolution, and multiphoton microscopy that offered excellent optical sectioning and signal-to-noise ratio. We systematically varied the concentration of two different odorants and found that responses in ORNs from *Ano2*^*−/−*^ animals were consistently larger at most concentrations. Interestingly, at lower concentrations of the two odors, the response amplitudes were similar between both groups. This result suggests that for low odor concentrations, ORN transduction currents may remain sufficiently modest, and limits further amplification through Ano2. In such a scenario, ORN transduction currents may be primarily carried through cyclic nucleotide-gated channels upstream of Ano2, thereby generating small membrane depolarizations without engaging significant Ano2-mediated amplification.

Biophysical studies *in vitro*, however, indicate that Ano2 currents are activated even for weak stimuli^10,36^. *In vitro* preparations allow for titration of odor concentrations for each neuron, thus allowing careful analysis of transduction currents in different response regimes, including threshold and sub-threshold responses. Perhaps glomerular imaging does not have enough sensitivity to detect responses to low concentrations of odors, and potential differences between *Ano2*^+/+^ and *Ano2*^*−/−*^ animals were missed. *In vivo*, threshold odor concentrations are likely to activate only a subset ORNs due to their dispersion throughout the nasal epithelium^37^. The population imaging approach used in our studies further decreases the likelihood of detecting low-level signals because any responses arising a small number of ORNs are averaged across all ORNs that terminate at a given glomerulus. An alternative approach may include sparsely labeling ORNs with Ca^2+^ indicators to allow for recordings of individual optically isolated axon terminals; however, to date there is no reliable method available for such an approach.

Another key result is that responses saturated at lower amplitudes in *Ano2*^+/+^ than in *Ano2*^*−/−*^ glomeruli, suggesting that the presence of Ano2 had a “clamping” effect, and its absence loosens the clamp to allow greater activity. Our findings and work of others^9^, suggest that in a high odor concentration regime, Ano2 may function as a feedback mechanism to limit the number of spikes generated by ORNs following odor stimulation. The proposed mechanism of action operates through a potent depolarization-induced inactivation of Na^+^ channels following transduction current amplification by Ano2^9^. A potential caveat is that the larger responses we observe in *Ano2*^*−/−*^ animals are the result of lower resting fluorescence due to a decrease in spontaneous ORN activity at some glomeruli^9^, thereby increasing the dynamic range available for Ca^2+^ indicator activity. Our experiments used odor concentrations that are generally thought to be sub-saturating for odor evoked responses in ORNs; however, it remains possible that at these concentrations, the largest signals observed in *Ano2*^+/+^ animals exceeded the range of our indicator due to a greater basal Ca^2+^ tone. Our results here argue against this possibility since the only observable differences occurred in response to strong odor stimulation – larger responses in *Ano2*^*−/−*^ animals would not be observed if ORN responses in *Ano2*^+/+^ were saturated at these higher concentrations.

It is also possible that Ano2 alters neural excitability in other ways, especially since Ano2 is expressed in ORN terminals^8,38^. For example, in the thalamocortical^39^ system Ano2 functions to suppress neural excitability by enhancing the magnitude of action potential after-hyperpolarization. In the hippocampus^40^, Ano2 decreases the duration of individual action potentials by relying on a chloride gradient that favors outward membrane currents close to resting potential^41^. Although the chloride gradient in the nasal epithelium favors inward currents at near resting potentials^16,18^, the presumed chloride gradient at ORN axonal terminals could yield outward currents through Ano2, triggered by inward flux of Ca^2+^ during action potentials. A reduction in ORN transduction currents at the olfactory cilia of *Ano2*^*−/−*^ animals could be offset by a reduction in the action potential after-hyperpolarization in the axonal compartments of ORNs.

Independent of the mechanisms involved, Ano2 seems to functionally compress the dynamic range of odor responses in individual ORNs by leaving weak responses unaffected (or enhancing them) and truncating the magnitude of responses to strong odors.

### Role of Ano2 in olfactory coding

Our data suggests that currents through Ano2 may serve as a negative feedback mechanism to prevent excessive activation at higher concentrations. It remains possible that at low to moderate concentrations, Ano2 may act to amplify sensory signals and affect activity in ways undetected by our measurements. For instance, the latency to first spike (from the onset of inhalation) could be shorter in *Ano2*^+/+^ ORNs because of the amplification by Ano2. Such changes in timing could play an important role in odor coding^42–44^, but our imaging methods may not have the temporal resolution or sensitivity to detect such differences in latency. It is apparent that simply scaling up the activity in ORNs is insufficient enhance odor detection and may instead have deleterious effects.

Another potential role of Ano2 may arise from differences in expression of Ano2 in ORNs of a common subtype. Through varying expression levels, ORNs projecting to the same glomerulus could de-correlate their firing patterns in response to the same stimulus by shunting their spike output at different levels and thereby increasing their information carrying capacity as a population. Past studies demonstrate that intrinsic biophysical diversity between sister mitral cells functionally reduces correlations in their spike output^45^ and these observations are consistent in other systems including ganglion cells^46^ and M1 type ganglion cell photoreceptors^47^ in the retina. In our mouse line, all ORNs were labeled with GCaMP3 and we were therefore unable to study heterogeneity at the single cell level. However, future studies may seek to record from a small number of ORNs projecting to the same glomerulus to determine whether Ano2 plays a role in de-correlating their spike output. Furthermore, a decreased information carrying capacity of ORNs in *Ano2*^*−/−*^ animals provides a potential explanation for the odor localization deficits observed in this study and others^9,10^. We observed that *Ano2*^*−/−*^ animals, despite having larger odor-evoked ORN responses, require longer to locate a relatively low-concentration odor source. While our result is in agreement with recent behavioral analyses of *Ano2*^*−/−*^ mice^9,10^, it stands in contrast to other reports that *Ano2*^*−/−*^ mice have no detectable deficits in odor discrimination^8^. Notably, our paradigm takes advantage of innate odor-seeking behavior in mice rather than task performance following learning. One possibility is that *Ano2*^*−/−*^ animals are able to overcome olfactory deficits through learning, thereby allowing for comparable performance when the experimental timescale is extended. Additional studies are necessary to explore potential differences in innate vs. learned olfactory behaviors, as well as any compensatory adaptations in *Ano2*^*−/−*^ mice.

Our result suggests that deletion of Ano2 does not simply scale up the sensitivity of ORNs, but rather, results in a fundamental reformatting of how odor information is transmitted to the brain. At present it is not clear how odor information is restructured in ORNs of *Ano2*^*−/−*^ animals, or if dysfunction in odor information processing is further compounded by downstream neurons.

## Materials and Methods

### Animal Care, General Statements

The *Anoctamin-2* knock-out (*Ano2*^*−/−*^) mouse line was obtained from the PBmice project of Fudan University (see Li *et al*., for characterization)^24^. C57BL/6 J, *Ano2*^+/+^, *Ano2*^*−/−*^, and OMP-GCaMP3^25^ mice were used in this study. The age of all animals at the time of the experiments was two to six months. All mice used in this study were housed in an inverted 12-hour light cycle and fed *ad libitum*. All the experiments were performed in accordance with the guidelines set by the National Institutes of Health and approved by the Institutional Animal Care and Use Committee at Harvard University.

### *In vivo* imaging

#### Surgery

Adult mice were anesthetized with an intraperitoneal injection of ketamine and xylazine (100 and 10 mg/kg, respectively) and eyes were covered with petroleum jelly. The scalp was shaved and opened. After thorough cleaning and drying, the exposed skull was gently scratched with a blade, and a titanium custom-made headplate was glued on the scratches. The cranial bones over the OBs were then removed using a 3 mm diameter biopsy punch (Integra Miltex). The surface of the brain was cleared of debris and a glass coverslip was glued into the vacated cavity in the skull. Dental cement (Jet Repair, Lang Dental) was used to cover the headplate and form a well around the cranial window. Animals were allowed to recover for at least three days. Prior to each imaging session, animals were anesthetized with a mixture of ketamine and xylazine (90% of dose used for surgery) and body temperature was maintained at 37 °C by a heating pad.

#### Epifluoresence

Two photo lenses coupled front to front were used to image the OB surface onto the sensor of a CMOS camera (DFK 23GPO31, The Imaging Source GmbH). Images (960 × 600 pixels) were acquired at 8-bit resolution and 8 frames/s. Data from the camera were recorded to the computer via data acquisition hardware (National instruments) and custom software in Labview. A blue LED (CBT-90, Luminus) with a maximum output of 1.65 mW/mm^2^ was used for excitation.

#### Multiphoton

A custom-built two-photon microscope was used for *in vivo* imaging. Fluorophores were excited and imaged with a water immersion objective (20× , 0.95 NA, Olympus) at 920 nm using a Ti:Sapphire laser (Mai Tai HP, Spectra-Physics). Images were acquired at 16-bit resolution and 4 frames/s. The pixel size was 1.218 μm, and fields of view were typically 365 × 365 μm. The point-spread function of the microscope was measured to be 0.51 × 0.48 × 2.12 μm. Image acquisition and scanning were controlled by custom-written software in Labview. Emitted light was routed through two dichroic mirrors (680dcxr, Chroma and FF555- Di02, Semrock) and collected by a photomultiplier tube (R3896, Hamamatsu) using filters in the 500–550 nm range (FF01–525/50, Semrock).

#### Odor stimulation

Monomolecular odorants (Sigma) were used as stimuli and delivered by custom-built 8 channel (epifluorescence experiments) or 16 channel (2-photon experiments) olfactometer controlled by custom-written software in Labview (National Instruments)^27^. Odorants were maintained at a nominal volumetric concentration of 16% (v/v) in diethyl phthalate and further diluted 8 times with air for a final concentration of 2% for epifluorescence imaging. For multiphoton imaging odors were diluted in mineral oil at 16% (v/v) and diluted 16 times with air for a final concentration of 1%. For most experiments, odors were presented for 5 s with an interstimulus interval of at least 40 s.

The odor panel for epifluorescence imaging consisted of 1) Methyl propionate 2) Methyl butyrate 3) Ethyl Valerate 4) Pentyl acetate 5) Propyl acetate 6) Valeraldehyde 7) Methyl tiglate.

The odor panel for multiphoton imaging consisted of 1) Ethyl tiglate 2) Ethyl valerate 3) Valeric acid 4) Allyl butyrate 5) Isoamylamine 6) 2-Methoxypyrazine 7) Isugenol 8) Methyl tiglate 9) Valeraldehyde 10) Isoamyl acetate 11) Heptanal 12) mineral oil 13) Ethyl propionate 14) Propyl acetate 15) 2-Heptanone 16) Acetophenone.

For imaging glomerular responses to odor concentrations an additional 16 channel olfactometer outfitted with two odors, Ethyl valerate and Allyl butyrate, was used. The initial concentration series for each odor was 80%, 16%, 8%, 1.6%, 0.8%, 0.16%, 0.08% (v/v) in mineral oil and further diluted 16 times with air. Odors were presented for 2 s to prevent adaptation at the strongest concentrations. For all experiments, odors were delivered 3–5 times each.

#### Analysis

Calcium signals were extracted from raw images using custom-written scripts in MATLAB (MathWorks Inc.) and reported as ΔF/F signals, where F represents the average baseline fluorescence. Regions of interest were selected from average fluorescence projections for multiphoton imaging and ΔF/F projections for epifluorescence imaging. Response amplitude was measured from between three and five repeats of each odor as the mean response in the 5 seconds following odor onset. For analysis of response kinetics, measurements of the response rise time and decay time constant were taken from individual trials rather than reported as the mean to capture any intertribal variability. Rise time was measured as the time from when the signal first deviated 3.5 standard deviations from the mean of the baseline period to the peak of the response. Decay constants were obtained by fitting a single exponential to the signal, starting at the peak. Bleaching was corrected by fitting a single exponential to blank odor trials in multiphoton imaging and fitting a single exponential to the baseline period for epifluorescence experiments. For images of ΔF/F signals, the mean of an equal number of median filtered frames in the baseline and odor period was used. Traces of ΔF/F signals were smoothed for display. For figures where a threshold was applied to the data, thresholds were calculated based on the distribution of blank odor trials. An area under the receiver operating curve analysis was performed and the lowest threshold yielding ten responses for every one blank odor response was chosen. Sparseness measures were calculated as previously reported^48,49^. Population sparseness measures the fraction of glomeruli that are activated by a given odor, with values near one indicating uniform activity across all glomeruli and values near zero indicating a lack of activity in most glomeruli:

1 $$P{S}_{j}=\frac{{({\sum }_{i=1}^{n}\frac{{r}_{i}}{n})}^{2}}{({\sum }_{i=1}^{n}\frac{{{r}_{i}}^{2}}{n})}$$

Where: $n$ = the number of glomeruli, ${r}_{i}$ = the response of glomerulus $A$ to odor $j$.

Lifetime sparseness measures the extent to which a given glomerulus responds to different odor stimuli. Values near one indicate all odors uniformly activate a given glomerulus and values near zero indicate a high degree of odor selectivity:

2 $$L{S}_{i}=\frac{{({\sum }_{j=1}^{m}\frac{{r}_{j}}{m})}^{2}}{({\sum }_{j=1}^{m}\frac{{{r}_{j}}^{2}}{m})}$$

Where: $m$ = the number of odors, ${r}_{j}$ = response of glomerulus $A$ to odor $j$.

All statistical comparisons for imaging experiments were made as described in the text for each figure and values are given as mean + /− standard error of the mean.

### Respiration measurements

#### Surgery

Animals were anesthetized with ketamine/xylazine as described above and a head plate was implanted in the skull as described previously in this article. For some mice, a small craniotomy was also made through the right nasal bone (1 mm anterior from the frontal/nasal fissure, 1 mm lateral from the midline), and a hollow cannula (#C313G; Plastics One Inc.) was lowered into the hole and glued to the skull. Finally, the whole exposed skull was covered with dental cement (Jet Repair, Lang Dental). The animals were given a week after the surgery to recover before any experiment was performed.

#### Respiration monitoring

Two strategies were used to monitor the breathing: measuring the intranasal pressure through an implanted cannula^50,51^, and measuring the temperature in front of the nose^52^.

For the intranasal pressure strategy, animals previously implanted with a cannula were head-fixed. Then, the cannula was connected to a pressure sensor (24PCEFJ6G; Honeywell International) through a piece of polyethylene tubing. The voltage signal generated by the sensor was amplified 1000×, low-pass filtered at 60 Hz, and digitized at 1000 Hz using custom software written in Labview.

For the temperature measurement, mice were head-fixed, and a thermocouple (5TC-TT-JI-40–1M, Omega Engineering) was placed ~2 mm in front of their nose. The voltage changes generated by the temperature variations were amplified 10000×, low-pass filtered at 60 Hz, and digitized at 1000 Hz using custom software written in Labview.

#### Analysis

Analysis of breathing signals and statistical tests were performed using custom software written in MATLAB. Two types of statistical tests were used: the Kruskal-Wallis test, and a bootstrap test to compare the means of two distributions (MATLAB function “bootstrp()” repeated 1,000,000 times for each bootstrapped statistics). The MATLAB toolbox CircStat was used to analyze circular data^53^. The critical p-value was set at 5% for all the tests, and Bonferroni correction was applied for multiple comparisons. On the figures, all the values are given as mean + /− standard error of the mean, unless otherwise stated.

### Open Field Behavior

The arena consisted of 56 cm diameter circular inner chamber with four air inlets equally spaced around its circumference. The circular inner chamber was housed in light- and sound-proof outer chamber and illuminated using infrared LEDs. Throughout each experiment, airflow was maintained at a constant velocity for each inlet. After 10 minutes baseline exploration, air to one of the inlets was redirected through an odorized chamber while ensuring no change in its velocity. A vacuum was located at the center of the arena and its flow matched to the sum of all air inlets to prevent the accumulation of odor in the arena. Peanut oil was diluted in mineral oil as in imaging experiments. Each animal was only tested once and the order in which they were tested was randomized. After each trial the arena was thoroughly cleaned with ethanol to eliminate the presence of social cues. Images for tracking were acquired at 8 Hz using a USB camera (Grasshopper3, Point Grey Imaging) and custom-written Labview software. Images were processed using custom MATLAB routines to measure location and velocity. Animals with an initial position >10 cm from the odor source were excluded from our analysis. All statistical comparisons for behavior experiments were made with Wilcoxon rank-sum test and values are given as mean + /− standard error of the mean.

### Data availability

All datasets in this manuscript are available from the corresponding author upon request.

## Electronic supplementary material

Supplementary Information
