# Supplementary material for: Calcium-activated chloride channels clamp odor-evoked spike activity in olfactory receptor neurons
Source: Sci Rep. 2018 Jul 13;8:10600. doi: 10.1038/s41598-018-28855-3 (PMC6045664; doi:10.1038/s41598-018-28855-3)
Supplement: Supplementary file 1 — Supplementary Information [file 41598_2018_28855_MOESM1_ESM.pdf]

Calcium-activated chloride channels clamp odor-evoked spike activity in olfactory receptor neurons

Joseph D. Zak[1], Julien Grimaud[1,2], Rong-Chang Li[3], Chih-Chun Lin[3], Venkatesh N. Murthy[1]*

1. Department of Molecular and Cellular Biology & Center for Brain Science, Harvard University, 16 Divinity Ave. Cambridge, MA 02138

2. Molecules, Cells & Organisms Program, Harvard University

3. Johns Hopkins University School of Medicine, Preclinical Teaching Building Room 905A, 725 North Wolfe Street Baltimore, MD 21205

*correspondence to vnmurthy@fas.harvard.edu

Supplemental Information

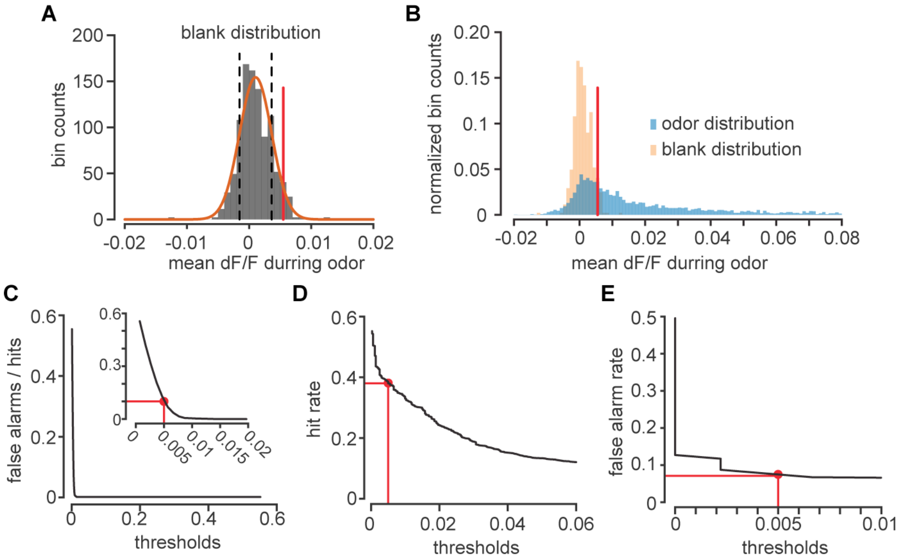

Supplemental Figure 1. Receiver operating characteristic threshold for wide-field imaging. **A**. Distribution of responses collected from no odor (blank) trials. Orange line is a plot of a probability density function with the same mean and variance as the collected data. Dashed lines indicate the first standard deviation from the mean. Red line is the ROC threshold. **B**. Distribution of odor trials overlaid on the distribution of no odor trials from part A. Red line is the ROC threshold. **C**. Curve of false alarms per hits for individual thresholds. Inset is an expanded curve. The threshold was selected to provide one false alarm per every ten hits. **D.** Plot of the hit rate as a function of threshold values. **E.** Plot of the false alarm rate as a function of threshold values.

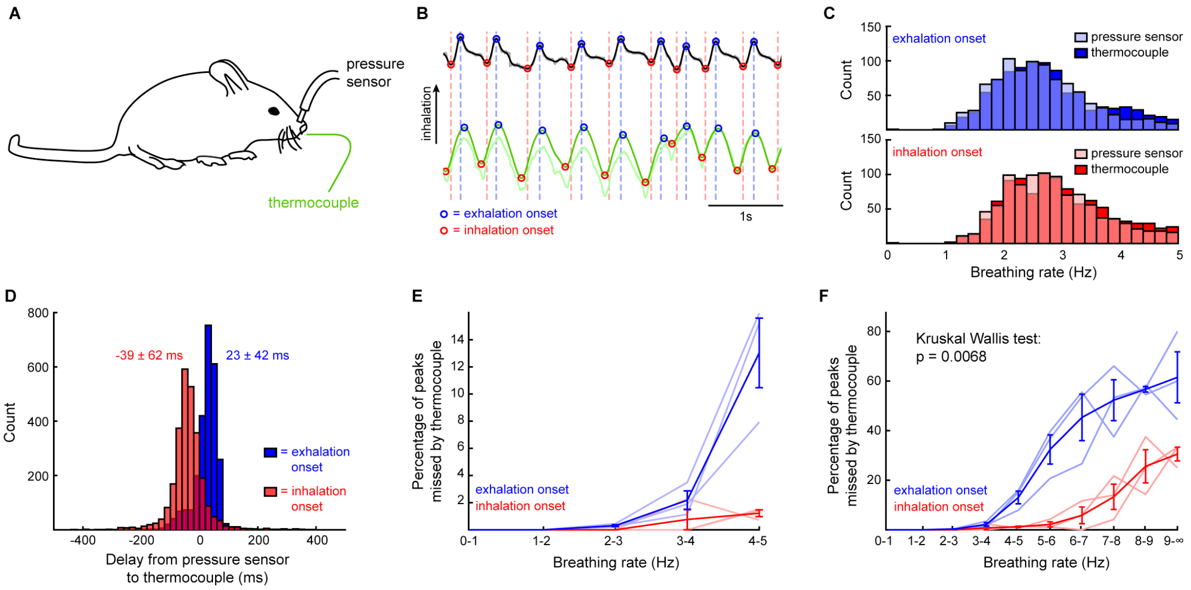

Supplemental Figure 2: Validation of the external thermal sensor versus pressure sensor for respiration monitoring. **A**. Experimental setup. The breathing of head-fixed, awake mice (n = 3) was monitored through two methods: a cannula implanted in the nasal cavity, connected to a pressure sensor, and an external thermal sensor (thermocouple) in front of the nostrils. **B**. Examples of respiration traces. Inhalation is upward. Light traces show raw data and dark traces are the data after digital filtering. Black: pressure sensor. Green: thermocouple. The blue dots show the peaks of inhalation, while the red ones show the peaks of exhalation. **C**. Histograms of the instantaneous respiration rate of an exemplar mouse using a pressure sensor (darker color) or the thermocouple (lighter color). Inhalation is the histogram on top in blue and exhalation is the histogram in red on bottom. **D**. Histogram of the delay between peak inhalation and exhalation measured from the pressure sensor and thermocouple across all respirations. Values next to each distribution: mean +/- standard deviation. **E**. Fraction of the inhalation or exhalation peaks missed by the thermocouple, as a function of the breathing rate. Each lighter curve is a different mouse. Each darker curve gives the mean +/- standard error of the mean. At respiration frequencies lower than 5Hz, which is typical of anesthetized mice, the thermocouple is a reliable method for the monitoring of breathing rates **F**. Graph from part E expanded to higher respiration rates. At respiration frequencies higher than 5Hz, which is only seen in awake mice, the thermocouple is not a reliable method for the monitoring of breathing rates (Kruskal Wallis test to compare the two distributions, p = 0.007).

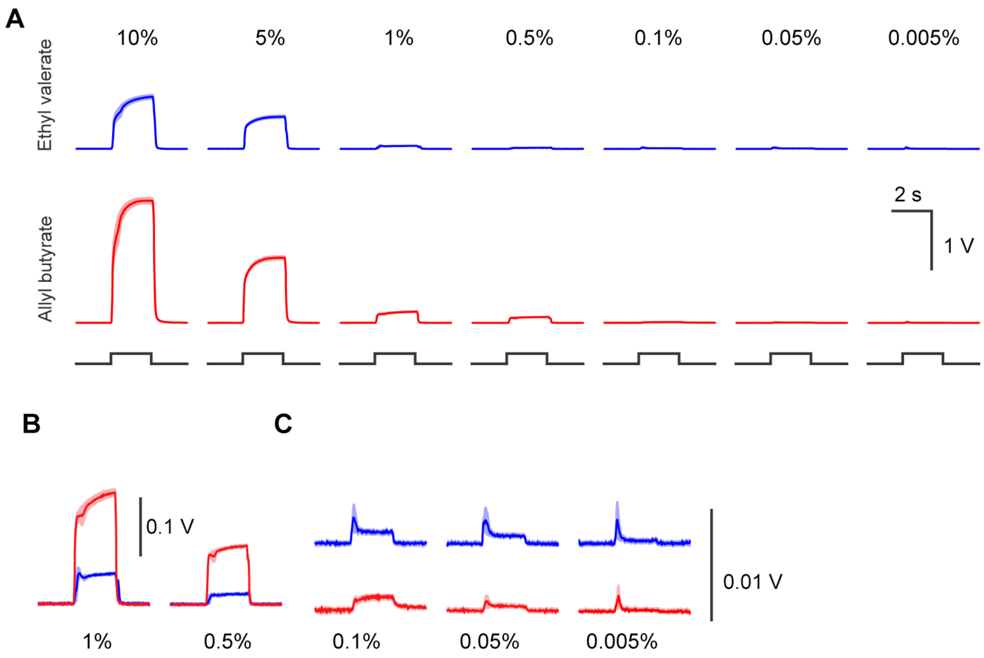

Supplemental Figure 3. Photoionization detector measurements of odor concentrations. **A**. Mean photoionization detector (PID) signals across five repeats for Ethyl valerate and Allyl butyrate. Shaded area each trace is the s.e.m. Black line below indicates the digital signal used to open each solenoid. **B**. Expanded and overlaid PID signals from odors measured at 1% and 0.5%. **C**. Expanded PID signals from odors at 0.1%, 0.05%, and 0.005%. Signals are expanded 100 times from part A.

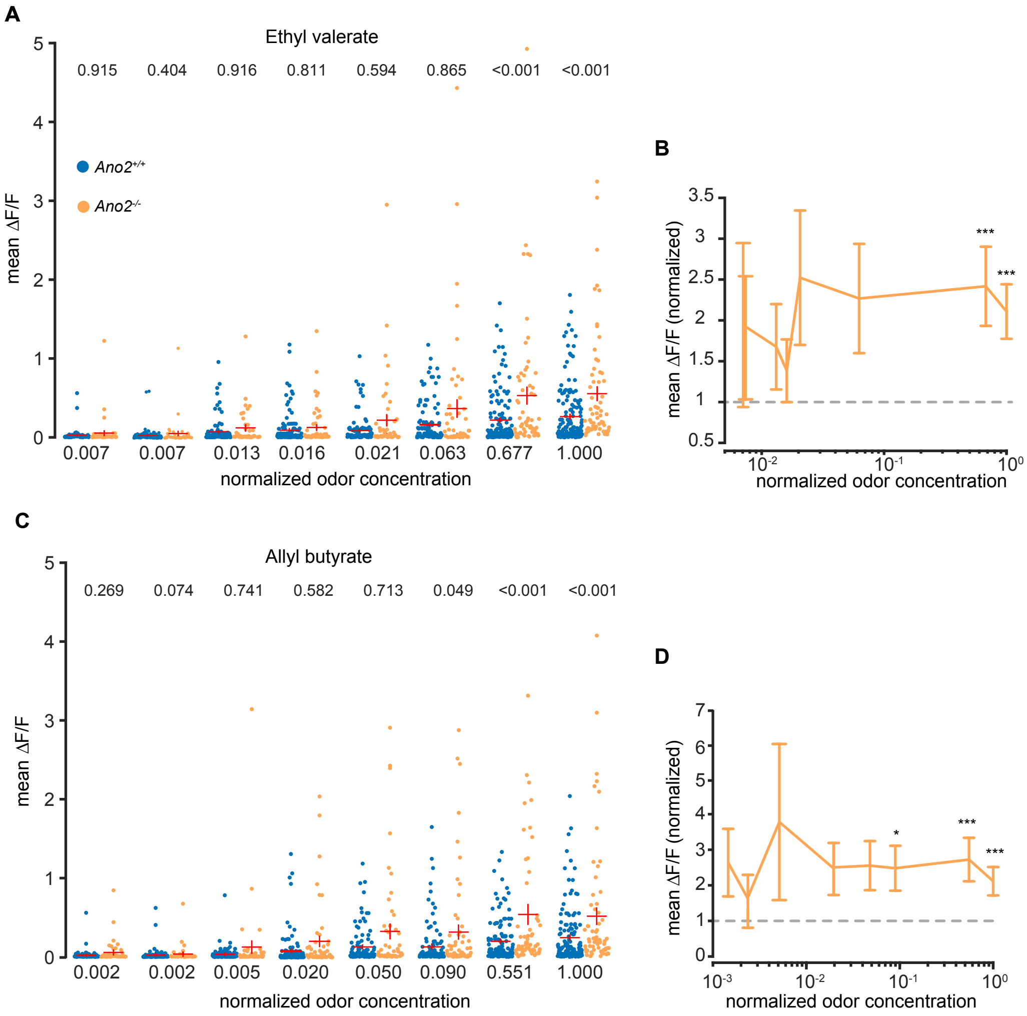

Supplemental Figure 4. Calcium responses at different concentrations for two odors. **A**. Responses of all glomeruli to Ethyl valerate. Red horizontal line indicates the mean and red vertical bars are the standard error. Normalized odor concentration is given below each scatter plot and the exact non-corrected p value obtained from a Wilcoxon rank-sum test is listed above each plot. **B**. Mean responses from *Ano2*$^{-/-}$ animals normalized to the mean response in *Ano2*$^{+/+}$ animals. Comparisons made with a Wilcoxon rank-sum test. **C-D**. Same as above for the odor Allyl butyrate.
